# Intralobar pulmonary sequestration: robotical resection using indocyanine green

**DOI:** 10.1093/jscr/rjad028

**Published:** 2023-03-03

**Authors:** Jonas Mohnke, Danjouma Cheufou Housmanou

**Affiliations:** Department of Thoracic Surgery, Clinical Centre Würzburg, Würzburg, Germany; Department of Thoracic Surgery, Clinical Centre Würzburg, Würzburg, Germany

## Abstract

Pulmonary sequestration (PS) is a relatively rare congenital lung anomaly. There are two different subtypes of PS, intralobar and extralobar sequestration. The majority of cases is constituted by intralobar sequestration. Here, we report a case of a 39-year-old female with intralobar sequestration, which was successfully resected by robotic-assisted surgery.

## INTRODUCTION

Pulmonary sequestration (PS) is a rare congenital malformation in the bronchopulmonary system. It is defined as an amount of nonfunctioning dysplastic lung tissue with an anomalous supply of arterial blood and a variety of venous drainage [1]. PS is a relatively rare condition comprising 0.15–6.4% of congenital lung malformations [2]. Since PS is considered a childhood disease, it is usually diagnosed at a young age and can be treated at an early stage. However, in some cases, asymptomatic PS can be found incidentally on computed tomography (CT) scan. There are two different subtypes of PS, intralobar and extralobar sequestration. The majority of cases is constituted by intralobar sequestration and is mostly located in the left lung [3]. The gold standard of therapy is the resection of the sequestrated lung [4]. Here, we report a case of a 39-year-old female with intralobar sequestration, which was successfully resected by robotic-assisted surgery.

## CASE REPORT

**Figure 1:**
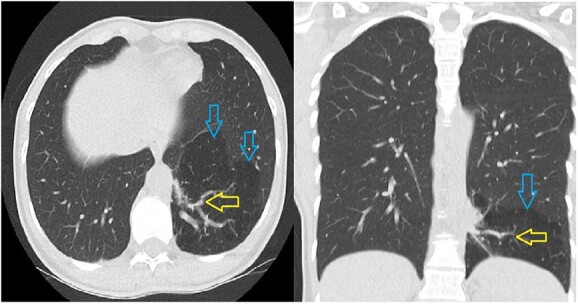
The CT scan shows a hypodense part in the left lower lobe (blue arrows) with an aberrant artery (yellow arrows) arising from the thoracic aorta.

The 39-year-old female patient was referred to us after a PS was found incidentally on a thoracic CT scan. This scan had been performed due to a suspected collagenosis, which was confirmed thereafter. However, PS was asymptomatic so far. Computed tomography showed a lesion of 8.7 cm × 9.8 cm × cc 7.7 cm not being in communication with the tracheobronchial system. An aberrant artery was branching from the thoracic aorta (Fig. 1). She was diagnosed with intralobar PS and minimally invasive surgery with the help of a robotic daVinci X System was indicated.

Under general anesthesia and double lumen intubation, robotic-assisted surgery was performed on the left side.

The surgical procedure was a 3-arm approach using a Fenestrated Bipolar forceps, a 30° lens and a Maryland forceps (Intuitive Surgical) without utility incision. After dissection of the triangular ligament, the aberrant artery branching from the thoracic aorta and the venous drainage in the hemiazygos vein were dissected.

**Figure 2:**
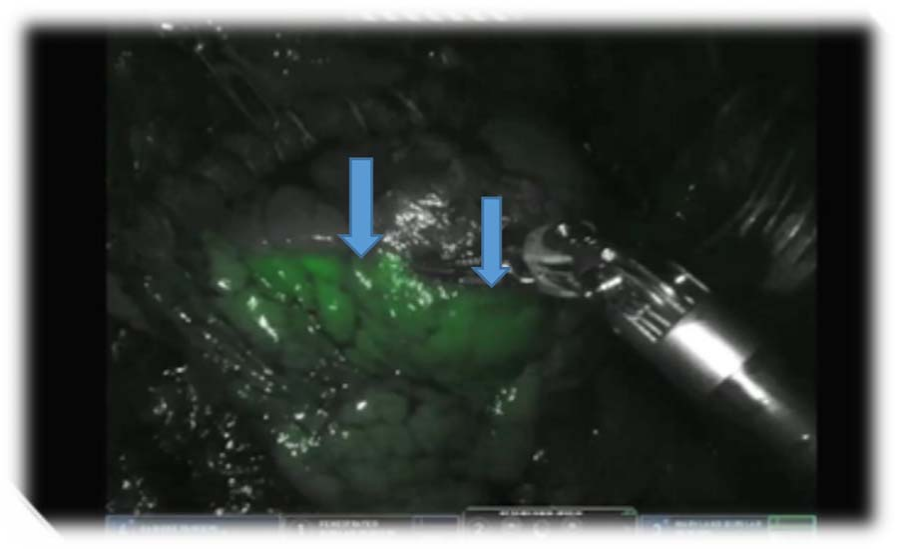
ICG showing the delineation of PS.

Further preparation clearly revealed the upper and lower lobe of the pulmonary vein. For a better visualization, indocyanine green (ICG) diluted in a concentration of 2.5 mg/ml NaCl solution was administered intravenously as a bolus by the anesthesiologist. We observed a good vascularization of the complete left lung using the real-time fluorescent firefly mode of the DaVinci system. The aberrant artery and vein were then divided using a 45 mm Da Vinci stapler. The reapplication of ICG showed a non-perfused PS (Fig. 2). We divided the PS from the normal lung tissue along the demarcating margin using a 45 mm Da Vinci Stapler. A single chest tube was placed at the end of the procedure and the postoperative clinical course was uneventful.

## DISCUSSION

Intralobar sequestrations are mostly treated with a lobectomy [5]. A sublobar resection should be preferred whenever possible to preserve lung tissue since malignancy is rare [6].

Recent publications have demonstrated the usefulness and safe applicability of ICG in lung sublobar resections with precise identification of the segmental margin. In our case, we injected 5 mg ICG and could visualize the margin between PS and normal lung tissue of the left lower lobe. Some authors described the successful use of 10 mg ICG for the same purpose [7, 8].

Thoracotomy has been used for better control of the aberrant vessel in case of intraoperative ruptures. The robotic-assisted resection, as previously demonstrated by numerous authors, provides an excellent three-dimensional view. The advantages of minimally invasive surgery have been well demonstrated [9, 10].

PS is often localized in the left lower lobe. However, some publishers mentioned the localization in the right lung, in both lungs or even combined in left thoracic and abdominal cavities [11, 12].

Clinical cases have been published describing the colonization of PS with aspergillus. In patients with asymptomatic PS discovered incidentally like in our patient, it is debatable whether a primary resection or further clinical observation should be performed. Elective surgery can be preferred to avoid conditions like bleeding or infection and to exclude the risk of malignancy [13].

The crucial part of the resection of a PS is the identification of the aberrant artery. In our case, the CT scan showed the artery branching from the thoracic aorta.

Following our experience, we suggest a minimally invasive approach using ICG to identify the exact boundary between PS and normal lung tissue.
